# Editorial: Paradoxes of diversity, equity and inclusion: from the lab to the social field

**DOI:** 10.3389/fpsyg.2024.1511223

**Published:** 2024-12-13

**Authors:** Claudia Toma, Smaranda Boroş, Maria Popa-Roch

**Affiliations:** ^1^Solvay Brussels School of Economics and Management, Université libre de Bruxelles, Brussels, Belgium; ^2^Vlerick Business School, Area People and Organisation, Ghent, Belgium; ^3^Department of Marketing, Innovation and Organisation, Faculty of Economics and Business Administration, Ghent University, Ghent, Belgium; ^4^Université de Strasbourg, Laboratoire Interuniversitaire des Sciences de l'Education et de la Communication, Strasbourg, France

**Keywords:** diversity, equity, inclusion, paradoxes, policies, diversity ideologies, continuum

Contemporary societies strive for multiculturalism and tolerance. To create conditions to reach this ideal, there should be a continuum between what the social actors are prepared for in school, the practices they encounter in the workplace, and the way they are welcomed (Roberson and Scott, 2024) and can contribute to the broader society. This continuum should be materialized in consistent conceptualizations and practices of diversity, equity, and inclusion (DEI) across educational, organizational, and societal contexts. However, what we see in practice (Post et al., 2021; Roberson, 2019) is fragmentation instead of continuum and consistency in focus and definition, with little dialogue between research and policy implementation and between research in educational and organizational environments. Inclusive education practices focus on students with special needs, with broader definitions of diversity being neglected. In organizations, the emphasis is mainly on diversity, inclusion and equity being more recent Research Topics and practices. Research conducted at the societal level addresses the comprehensive ideologies underlying diversity and inclusion (Konadu-Osei et al., 2023). This insufficient conceptualization within and across domains gives rise to the many paradoxes we see in the research and praxis of DEI.

This Research Topic is our invitation to a dialogue that builds bridges between the various types (fundamental vs. applied) and domains of research (educational, organizational, and societal) to contribute to moving the field of DEI forward. In this editorial paper, we point to various paradoxes within and between domains by analyzing the focus of DEI policies and practices and the conditions that make them effective. We aim to gain clarity and continuity across fields at the conceptual, empirical, and applied levels.

In the educational domain, the paper of Buchs et al. highlights the necessity of having inclusive programs that broaden their scope by moving from the traditional focus on students with disabilities to other forms of disadvantage, such as linguistic-related status. Although cooperative learning effectively supports inclusive education (it fosters positive relationships and facilitates learning for all), it is rarely implemented. Authors point to the paradox that not all-inclusive programs benefit disadvantaged students, while some even reinforce inequalities between high and low-status students by exacerbating the achievement gap. To counteract the adverse effects, they propose using cooperative learning with a high level of implementation that sustains equal participation of all and ensures positive intergroup contact between students from different linguistic groups. Their results show that an inclusive program based on multilingual cooperative activities positively impact students with a low status. Another reason why DEI policies in education are ineffective is because they focus on isolated identity experiences (e.g., based solely on gender, social class, or ethnicity) and do not recognize the complex system of disadvantage and exclusion through an intersectional approach. The paper of Fernandez et al. points to this paradox in a higher education context related to universities' diversity and inclusion strategies. Along with the necessity to consider the role of the intersection of social class with other identities, policies should consider the needs and viewpoints of disadvantaged students from a bottom-up perspective based on institutional change and less on the individuals' capacities to cope with institutional norms. DEI initiatives should be founded on the participants' expertise in making sense of their experience to avoid being disconnected from the individual and group needs.

A critical challenge in the DEI domain is conceptualizing the notion of disadvantage by defining what diversity means. The paper of Zhang and Kirby demonstrates a shift in diversity definitions to include fewer protected demographic groups and more non-demographic characteristics, particularly among dominant group members with anti-egalitarian and colorblind belief systems. Thus, while the research suggests the necessity to broaden and complexify the notion of disadvantage, advantaged individuals are motivated to move the focus of DEI from characteristics that create systemic inequalities to characteristics that refer to any form of difference. This also suggests that advantaged individuals perceive the DEI initiatives that aim to reduce inequalities and create more inclusive environments for the disadvantaged as threatening. Therefore, paradoxically, barriers for disadvantaged groups will disappear to the extent that barriers for the advantaged are removed, too. Intersectionality may be a solution here as, for example, not all men are privileged in terms of ethnicity, social class, physical ability or sexual orientation. The recommendation proposed by the paper of Van Laar et al. is to make advantaged group members allies of DEI policies, as they are pivotal agents for change in work organizations, education, and society. With a focus on gender equality policies, the authors show that men's privileged status is potentially threatened by progress in gender equality, with negative consequences on these gender-equality initiatives in a vicious circle. At the same time, they highlight how men themselves are victims of restrictive gender roles, with negative implications for health, risky behavior, wellbeing, and work outcomes. Thus, the threat elicited by DEI practices among majorities represents a significant challenge to make progress with DEI. The authors provide paths to men's involvement in gender equality progress, which may inspire striving for equality in other diversity domains.

The idea of DEI threat among the majority groups is also supported by the paper of Andriessen et al., who bring empirical support from a national survey in the Netherlands. The authors show that perceptions of inclusion climate have opposite effects on the minority and majority. When the majority group perceives the national climate to be more inclusive toward minorities, they report higher levels of ethnocentrism and avoid direct inter-ethnic contact. The opposite is found among the minority group with improved feelings of belonging, participation, and positive intergroup attitudes. Paradoxically, for both minority and majority groups, the perception of an inclusive climate predicts opposition to increased ethnic diversity. This suggests that the relationship between diversity and inclusion is not straightforward and that some DEI practices and contexts allow a positive relationship while others trigger a negative one. In the context of diversity and inclusion in work teams, De Saint Priest et al. have shown that statements promoting diversity value in organizations lead team members to choose more diverse teams but fall short of actual inclusion. In their paper, they examined if the organizations' statements reflecting the commitment to age diversity and fair treatment of mature workers increase representation and inclusion of older people. The authors find evidence that diversity statements increase the representation of older employees in teams but that it does not trigger inclusive behavior. Having broad diversity statements without explicit reference to inclusion may not be enough. This effect may not be limited to age diversity. Diversity statements may lead to paradoxical unintended effects. Therefore, individuals are willing to select diverse teams and behave inclusively toward new team members only when the organizational rationale underlying diversity statements is to change toward a more inclusive workplace. Managers' behaviors are essential in achieving organizational change and dealing with DEI resistance. The paper of Boroş and Gorbatai calls attention to the characteristics that allow middle managers to implement organizations' DEI strategies. Their paradox mindset skills (acknowledging and adapting to the ongoing tensions of conflicting demands rather than trying to eliminate them) and emotional capabilities (the ability to recognize and understand emotions and to influence which emotions they have, when they have them, and how they experience and express these emotions) are crucial preconditions for the successful implementation of these strategies. A paradox mindset enables managers to reconcile the tensions inherent in DEI implementation, while emotional capabilities allow managers to effectively navigate the complex emotional dynamics elicited by diversity, thus contributing to more effective DEI policies.

Among the conditions that make DEI policies effective, the rationale and underlying diversity ideology promoted by organizations are other vital factors. Russell Pascual et al. analyzed diversity ideologies promoted by US universities and organizations to understand their nuances. They found that universities frame diversity ideologies regarding value-in-equality and use the moral and business rationale equally. In contrast, companies focus on value-in-individual differences and use the business case substantially more. However, because those ideologies reinforce a moralistic or instrumental value of diversity, they fall short of building a stronger case at the societal level, namely valuing group differences and positive inter-group contact. This paradox is also highlighted in the paper of Bosch. She shows that while the ultimate goal of DEI policies and practices is to achieve justice, organizations focus exclusively on attaining organizational justice in a simplistic and conflicting manner. Workplace DEI is subject to fashionable rhetoric and does not consider the complex nature of justice at work, avoiding the paradoxical ideas regarded as burdensome. To increase political gains, managers can claim ‘embracing people that are different to you, else you are bigoted' without the necessary attention to clashing values, beliefs, and cultures. The elucidation of the inherent paradoxes within DEI (i.e., of needs, of social value, of productive economy, of time) as experienced by the employers may result in less rhetoric and more thoughtful approaches to DEI. The solution is to broaden the scope of DEI policies to achieve social justice within both organizations and social systems. The paper of Zubareva and Minescu perfectly illustrates how a lack of focus on social justice can have disastrous consequences for diversity and integration policies at the societal level. Using the case of a protection directive (temporary protection directive to protect individuals fleeing the Russian invasion of Ukraine) that allowed Ireland to welcome Ukrainian refugees, they showed how this directive was poorly implemented as a policy, leading to more exclusion, prejudice, and discrimination toward the Ukrainian refugees. Simultaneously, they highlight the double standards responses and differential treatment of Ukrainian refugees in comparison with other immigrants and refugees, such as African, Middle Eastern, Roma, who faced important challenges. This gap between the policy intentions and their actual implementations is one of the paradoxical effects of DEI policies, which sometimes fail to achieve social justice and reduce inequality.

The papers published in this Research Topic suggest that DEI policies implemented in schools, organizations, and society have mixed and paradoxical results. This is partly because these policies' focus is unclear; they lack a clear conceptualization of the DEI notions and fail to consider essential conditions that make them effective. Another important reason is the lack of continuity and consistency across levels and in time: the focus of these policies in school is disconnected from the one in organizations and the one in society in general. Starting from the paradoxes the papers in this Research Topic call attention to, we propose future research directions and questions that would address these paradoxes in an insightful manner.

We acknowledge that paradoxes might exist within and between domains but they are inherent to all DEI policies and are at the root of new developments. One paradox identified both in education and organizations is the gap between what organizations/schools (see Rohmer et al., 2022 for school inclusion) say about DEI, what they do, and what they achieve, also called in the literature diversity decoupling (Toma et al., 2023; De Cock et al., 2024). Future studies should investigate if exposure to diversity decoupling is conducive to other paradoxes, such as DEI policies reinforcing inequalities and discrimination toward disadvantaged groups (see Boroş, 2022 for an example). A second paradox highlighted by the papers in this Research Topic is the increased resistance toward DEI policies and the necessity to bring the advantaged groups on board to make progress with DEI. Future research should focus on allyship dynamics and investigate the role of individual, group, and organizational-level processes to understand better when and why allyship in schools and organizations leads to positive outcomes. In addition, research should disentangle the immediate resistances and paradoxes from the more long-term ones and the associated costs. A third paradox is the lack of coherence between DEI policies at educational, organizational, and societal levels, and we call for research using longitudinal data or diary studies that investigate people's paradoxical experience with DEI policies in their different roles or varying stages of their professional and private lives. In sum, while most paradoxes highlighted in this Research Topic are detrimental to DEI progress, we encourage various actors in charge of DEI to acknowledge that paradoxes are ingrained and necessary to make progress with this complex endeavor.
